# Diagnostic methods for protozoan diseases: a review focused on leishmaniasis, Chagas disease and malaria

**DOI:** 10.3389/fmicb.2026.1735371

**Published:** 2026-02-23

**Authors:** Ada da Silva Matos, Rodrigo Nunes Rodrigues-da-Silva, Thais Stelzer Toledo, Laura Sant'Anna Ataides, Natália Debize da Motta, Cinthia Magalhães Rodolphi, Isabela Ferreira Soares, Francini Neves Ribeiro, Ana Luiza Carneiro Alencar, Fernanda de Moraes Maia, Josué da Costa Lima-Junior, Fernanda Nazaré Morgado

**Affiliations:** 1 Immunoparasitology Laboratory, Oswaldo Cruz Institute (IOC), Oswaldo Cruz Foundation (Fiocruz), Rio de Janeiro, Brazil; 2 Hantaviruses and Rickettsioses Laboratory, IOC, Fiocruz, Rio de Janeiro, Brazil

**Keywords:** Chagas disease, diagnosis, leishmaniasis, malaria, protozoan, review

## Abstract

Protozoan diseases remain a serious public health challenge, particularly in countries such as Brazil, whose continental dimensions and diverse ecological settings allow for multiple transmission cycles, involving a wide range of vectors, reservoirs, intermediate and definitive hosts, suitable habitats, and a complex socioeconomic context that increases exposure to various diseases due to social vulnerability. Consequently, diseases such as malaria, leishmaniasis, and Chagas disease are highly prevalent in Brazil, affecting a significant portion of the population, especially in regions marked by greater social inequality. In this context, this study aims to present the epidemiological landscape of these diseases and discuss the role of immunological and molecular diagnostic tools, as well as the fundamental concepts that are essential for evaluating these diagnostic approaches. Overall, this review provides a detailed summary of established diagnostic approaches of these diseases and emphasizes the integration of clinical and epidemiological information with the application of sensitive and specific diagnostic techniques aimed at promoting early detection, monitoring, and control of infections in endemic areas, highlighting the important role of diagnosis as a strategic tool in public health.

## 1 Introduction

Infectious diseases caused by parasites have rapid transmission and may involve human-to-human transmission, transmission through water or food resources, and may or may not require vectors (Johnson et al., 2015; Boucher et al., 2009; McDonald et al., 2018). Moreover, they represent an important cause of global morbidity and mortality (GBD 2013 Mortality Causes of Death Collaborators, 2015). According to the World Health Organization (WHO), they are the second leading contributor to human deaths, after cardiovascular condition (Abat et al., 2016). Within this context, there are the neglected tropical diseases (NTDs), which can be defined as those caused by infectious agents or parasites endemic to populations in situations of socioeconomic vulnerability, and which receive limited investment in research, drug development, and control and prevention measures (Engels et al., 2021). Some common neglected parasitic diseases in Brazil include Chagas disease and leishmaniasis (cutaneous and visceral) (Schröder et al., 2023).

The WHO estimates that NTDs affect more than 1 billion people and cause 120,000 deaths, with nearly 1.5 billion requiring preventive and/or curative interventions for NTDs (World Health Organization, n.d.(b)). In Brazil, it is estimated that around 30 million people may be at risk of contracting NTDs. Many NTDs involve vector-borne transmission and animal reservoirs, and furthermore, they are considered to result from processes of inequality and vulnerability that primarily affect tropical and subtropical areas, leading to physical disability, stigma, social exclusion, and premature deaths (Ministério da Saúde, 2024b).

In Brazil, almost all municipalities reported at least one case of NTD between 2016 and 2020, with an increase in cases of overlap (defined as three or more diseases occurring simultaneously), mainly impacting the North, Center-West, and Northeast regions (Ministério da Saúde, 2024b). It is also important to highlight that factors such as poverty, lack of investment in public education policies, and socioeconomic development contribute to the increase and persistence of NTDs in these regions (Rocha et al., 2023).

So, the study of epidemiology is essential for understanding the distribution of diseases and their determinants in the population, as well as playing a central role in prevention by identifying causes and modes of transmission. In this context, the epidemiological triad—composed of the etiological agent, host, and environment—serves as an important model for understanding how the interaction among these elements sustains transmission, which is influenced by biological, social, and environmental factors. The use of epidemiological indicators, such as morbidity and mortality rates, makes it possible to assess the population's health status and identify the groups most vulnerable to infection. This guides the adoption of prophylactic measures—such as vaccination, basic sanitation, and the promotion of healthy habits—along with strategies to prevent disease worsening, including early diagnosis and appropriate treatment (Neves, 2022).

**Figure 1:**
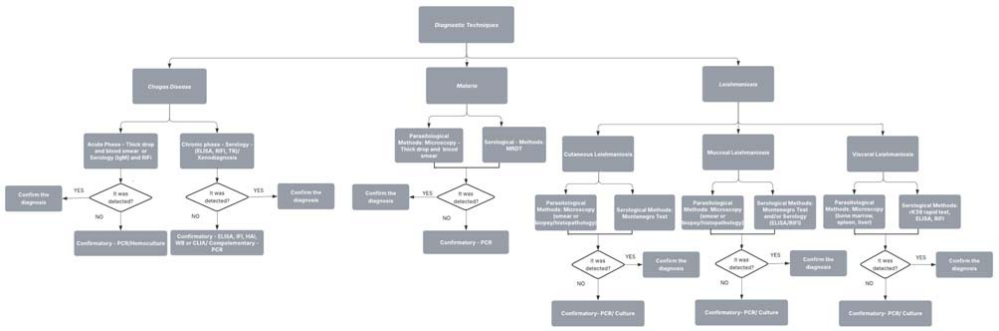
Summary of the diagnostic techniques used in the diagnosis of Chagas disease, malaria, and leishmaniasis. For Chagas disease, parasitological methods (thick drop and blood smear) are used in the acute phase, while serology and xenodiagnosis are applied in the chronic phase, with PCR or Western blot as confirmatory tests. For malaria, diagnosis relies on thick drop and blood smear microscopy or serology, followed by PCR confirmation. For leishmaniasis, diagnostic strategies include serology (TR and ELISA), lesion or biopsy evaluation by immunohistochemistry (IHQ), and alternative or complementary methods such as blood and bone marrow PCR.

In this context, tests based on the principle of antigen–antibody interaction, such as Enzyme-linked immunosorbent assay (ELISA) and Enzyme-Linked Immunospot (ELISPOT), Immunohistochemistry, Immunofluorescence and Flow Cytometry, as well as molecular tests such as Polymerase Chain Reaction (PCR) and Western-Blotting, represent two main diagnostic approaches with broad application in the diagnosis of protozoan diseases (Castellanos-Gonzalez et al., 2018; Marchiol et al., 2023), as shown in the flowchart shown in Figure 1, an explanatory summary of the diagnostic techniques used in the diagnosis of Chagas disease, malaria, and leishmaniasis. The aim of the manuscript is to discuss the importance of knowing the diagnostic tests available for each disease and their applicability at each stage of clinical care. Understanding how the methods work is also important for the correct interpretation of results, which directly impacts treatment, clinical follow-up, and outcomes.

## 2 Epidemiology of leishmaniasis, Chagas disease and malaria

### 2.1 Cutaneous and visceral leishmaniasis and mucocutaneous leishmaniasis

Leishmaniasis is a group of diseases caused by parasites from over 20 *Leishmania* species, which present two main clinical forms: cutaneous leishmaniasis (affecting the skin and/or mucosa) (CL) and visceral leishmaniasis (VL), affecting the lymphohematopoietic system. Leishmaniasis represents a significant global health challenge, primarily affecting the Americas, East Africa, North Africa, and West and Southeast Asia (SAÚDE OO-OP-AD, 2021), and is endemic in 90 countries.

CL is a non-contagious infectious disease that causes ulcers on the skin and/or mucosa and is transmitted to humans through the bite of infected female sandflies of the genus *Lutzomyia*, commonly known as sandflies (Silva et al., 2021). In Brazil, the main *Leishmania* species responsible for infection are *Leishmania* (*Leishmania*) *amazonensis, L. (Viannia) guyanensis*, and *L. (V.) braziliensis*. However, other species also occur, such as *L. naiffi, L. lainsoni, L. lindenbergi*, and *L. shawi*, which are also found in Brazil. WHO currently estimates that more than 1 billion people live in endemic areas and are at risk of infection, with approximately 30,000 new cases of VL and 1 million new cases of CL occurring annually (World Health Organization, 2025a). In Brazil, an average of approximately 21,000 cases is reported annually, with the North region exhibiting the highest incidence rate (46.4 cases per 100,000 inhabitants), followed by the Center-West (17.2 cases per 100,000 inhabitants) and Northeast (eight cases per 100,000 inhabitants) (Ministério da Saúde, 2025b).

In the Americas, VL is characterized as a chronic zoonotic disease that can involve systemic manifestations and may be fatal in up to 90% of cases if not properly treated. VL is also transmitted to humans through the bite of infected female sandflies of the genus *Lutzomyia*, with *Lutzomyia longipalpis* being the primary species involved. Transmission occurs when the female sandfly feeds on infected dogs (urban reservoirs) or wild animals (sylvatic reservoirs) and subsequently bites a human (Ministério da Saúde, n.d.(c)). It is estimated that 50,000 to 90,000 new cases occur worldwide each year, with the majority in East Africa, India, and Brazil (World Health Organization, 2023a). In Brazil, an average of approximately 2.000 new cases is reported annually, particularly in the North and Northeast regions, caused by *Leishmania infantum* (Marcondes and Day, 2019; Lopez et al., 2025).

The transmission cycles of CL and VL share similar characteristics, involving a mammalian host and a vector. During the blood meal, the sandfly injects infective promastigote forms into a susceptible mammal. These promastigotes are phagocytosed by macrophages and other phagocytic cells, transforming into amastigote forms that multiply by binary fission within a parasitophorous vacuole until causing cell lysis and subsequent phagocytosis by new cells, leading to eventual parasitemia (Esch and Petersen, 2013).

Infection of sandflies occurs through a blood meal taken from a host, ingesting infected macrophages. Once inside the insect vector, the amastigotes transform into promastigotes in the midgut, reproduce, and subsequently migrate to the valve stomodeal, where they develop into infective metacyclic promastigotes until the next blood meal (Esch and Petersen, 2013). It is noteworthy that domestic dogs are considered the primary urban reservoir of VL, whereas foxes and marsupials serve as sylvatic reservoirs, and generally, cases in dogs precede human cases (Costa et al., 2018; Brasil MdS, 2014; Camargo and Langoni, 2006).

The symptoms of CL are lesions on the skin and/or mucosa, which may be single, multiple, disseminated, or diffuse, typically presenting as painless ulcers (Ministério da Saúde, n.d.(b)). In contrast, VL is characterized as a systemic infection, causing prolonged fever, hepatosplenomegaly, weight loss, weakness, muscle fatigue, anemia, leukopenia, thrombocytopenia, hemorrhagic phenomena, among other clinical signs (Ministério da Saúde, n.d.(c)).

In humans, the pharmacological treatment of visceral and cutaneous leishmaniasis includes pentavalent antimonials (Pentostam^®^ and Glucantime^®^), amphotericin B, paromomycin, and miltefosine (Santos et al., 2023). In Brazil, miltefosine is the only drug administered orally and is currently also used for the treatment of canine visceral leishmaniasis, reducing the parasite load in the skin and bone marrow and it can also be combined with allopurinol to enhance the therapeutic response (Vaz et al., 2023). However, these treatments have limitations, including high cost, parasite resistance, therapeutic failure and severe adverse effects (Santos et al., 2023).

Accordingly, new orally administered treatment regimens are being investigated, including compounds such as 2,4,5-trisubstituted benzamides, 4,7,9-trisubstituted benzoxazepines, and farnesol (Kadayat et al., 2024; Kim et al., 2023; Sharma et al., 2023).

The 2,4,5-trisubstituted benzamides are benzamide derivatives that exhibit *in vitro* activity against *L. mexicana* amastigotes (EC_50_ = 0.66 μM) and display high oral bioavailability (80%) (Kim et al., 2023; Sharma et al., 2023). 4,7,9-trisubstituted benzoxazepines, derived from 4-[(3,5-dimethyl-4-isoxazolyl)acetyl]-9-[(1-methyl-3-piperidinyl)methoxy]-7-(5-methyl-2-thienyl)-2,3,4,5-tetrahydro-1,4-benzoxazepine, showed an EC_50_ of 2,3 μM against *L. mexicana* amastigotes and demonstrated good aqueous solubility (Kadayat et al., 2024). Farnesol, a sesquiterpene found in plants such as citronella, cyclamen, balsam, and musk, as well as in various essential oils, and known for its low toxicity, exhibited inhibitory dose-dependent activity against *L. major* promastigotes (EC_50_ = 175.7 μM) and amastigotes (EC_50_ = 945 μM), with a selectivity index (SI = 5.65). Its mechanism of action includes induction of apoptosis and inhibition of the enzyme lanosterol 14-demethylase, ultimately disrupting ergosterol biosynthesis (Kim et al., 2023; Sharma et al., 2023).

Vaccine development is challenging due to the complex life cycle of the parasite. The Sucrose Non-Fermenting 1 (SNF1) kinase protein has emerged as a potential target for vaccine development. Deletion of this gene using the CRISPR-Cas9 technique resulted in reduced promastigote growth, a lower proportion of metacyclic forms, and morphological deformation of mitochondrial cristae. In mouse models, this deletion also led to a reduction in lesion size (Shoeran et al., 2025).

Diagnosis of CL is performed using direct parasitological methods, such as lesion edge scrapings or imprint biopsy techniques. Histopathological examination, parasite isolation in culture, serology, and PCR can also be used (Xavier et al., 2006; Saldanha et al., 2017). VL diagnosis is also conducted via parasitological methods through the visualization of amastigote forms in collected material. According to the Brazilian Ministry of Health, bone marrow aspirate is considered the biological material of reference for direct parasitological diagnosis. However, other samples can be used to visualize *Leishmania* amastigotes, such as lymph nodes and spleen, which require procedures to be performed in a hospital setting and under surgical conditions. Other tests for the diagnosis of LV include: serology, in addition to PCR or rapid immunochromatographic tests (Gao et al., 2015; Sakkas et al., 2016).

Both forms of leishmaniasis can be controlled and prevented through vector management, including practices such as cleaning yards and vacant lots, removing organic material, using insecticides and repellents in areas with high case numbers, and implementing surveillance and environmental education measures in affected regions (World Health Organization, n.d.(c)).

### 2.2 Chagas disease

Chagas Disease, also known as American trypanosomiasis, is an infection caused by the protozoan *Trypanosoma cruzi*. It presents an acute phase, which may be symptomatic or asymptomatic, and a chronic phase, which can be asymptomatic or manifest with cardiac and/or digestive clinical symptoms (Ministério da Saúde, n.d.(a)). WHO estimates that approximately 7 million people worldwide are infected with *T. cruzi*, with around 10,000 deaths reported in 2017. Additionally, the disease has been detected in 44 countries (World Health Organization, n.d.(a)). In Brazil, during the period from 2023 to 2024, 5.460 cases of chronic Chagas disease were reported across 710 municipalities, with most cases occurring in urban areas (Ministério da Saúde, 2024a).

The transmission cycle of Chagas disease begins with the vector, an infected triatomine bug, during a blood meal on a host. The insect vector releases trypomastigote metacyclic forms in the feces near the bite site, which causes a minor injury, allowing the trypomastigotes to enter the host and invade nearby cells, differentiating into intracellular amastigotes. These amastigotes multiply by binary fission and subsequently differentiate into trypomastigotes, which are released into the bloodstream to infect new cells, transforming back into intracellular amastigotes. The triatomine becomes infected when feeding on a host with parasitemia (Prevention CfDCa, 2021).

It is noteworthy that Chagas disease can be transmitted through several routes, the main ones being: vector-borne, via direct contact with triatomine feces; oral, through ingestion of food contaminated with *T. cruzi*; vertical, via transmission from mother to baby during pregnancy or childbirth; or through blood transfusion, organ transplantation, and, less commonly, accidental contact of wounds or mucosa with contaminated material (Ministério da Saúde, n.d.(a)).

The most common symptoms of Chagas disease during the acute phase include prolonged fever lasting more than seven days, headache, severe weakness, swelling of the face and legs, and the appearance of a chagoma, the classical lesion of the disease, which resembles a boil at the site of the triatomine bite. Another characteristic symptom of the acute phase is Romaña's sign, a classic indicator of acute Chagas disease. After the acute phase, if the individual does not receive timely and appropriate treatment, the chronic phase of the disease may develop, initially remaining asymptomatic. However, over the years, infected individuals may develop cardiac, digestive, or cardio digestive complications (Ministério da Saúde, n.d.(a)).

Diagnosis of Chagas disease is based on the presence of suggestive signs and symptoms and an epidemiological history compatible with exposure to outbreaks. In the acute phase, diagnosis is generally performed through direct parasitological methods such as thick blood smear and peripheral blood smear and/or detection of anti-*T. cruzi* IgM antibodies. In the chronic phase, diagnosis is performed through the detection of antibodies using indirect immunofluorescence, hemagglutination, and ELISA assays, as well as DNA detection by PCR. The establishment of cell culture (blood culture) can also be employed (Souza and Amato Neto, 2012; Ramírez et al., 2009; Duarte et al., 2014).

In Brazil, for the treatment of Chagas disease, the first-line therapy is benznidazole, administered in two doses per day. As a second-line therapy, nifurtimox is recommended, administered in three doses per day for up to 60 days (Altcheh et al., 2025; Freire et al., 2025; Díaz-Menéndez et al., 2025). These drugs are capable of reducing parasitemia, and when cure is achieved, seroconversion occurs, with antibody levels becoming seronegative (Altcheh et al., 2025).

Benznidazole is effective in the acute phase of the disease; however, prolonged treatment allows the elimination of the bloodstream forms and reduces the tissue form of the parasite (Freire et al., 2025). This drug presents, as a disadvantage in the chronic phase, a reduced ability to eliminate amastigote forms, and it also has several side effects, such as neurological, dermatological, and gastrointestinal alterations, sometimes requiring treatment discontinuation. However, new drugs and therapeutic combinations have been studied (Díaz-Menéndez et al., 2025). The combination of benznidazole with curcumin showed a cure rate of 83.3%, while amiodarone and atorvastatin resulted in reduced parasitemia in the blood and demonstrated important cardioprotective effects in heart failure and arrhythmias (Freire et al., 2025).

### 2.3 Malaria

Malaria is an infectious disease caused by parasites of the genus *Plasmodium* and is transmitted to humans through the bite of infected female mosquitoes of the genus Anopheles, which are most active at dusk and dawn (Ministério da Saúde, n.d.(d)). In Brazil, the most important *Plasmodium* species are *P. malariae, P. vivax*, and *P. falciparum*, while *P. ovale* can occasionally be diagnosed in the country but is more frequently found in Africa (Oliveira-Ferreira et al., 2010; Limongi et al., 2014; da Silva, 2010). Among these species, *P. falciparum* is considered the most lethal and is the predominant species in Africa, while *P. vivax* predominates in most countries outside sub-Saharan Africa (Prugnolle et al., 2011; Howes et al., 2016).

According to WHO, an estimated 263 million malaria cases occurred worldwide in 2023, resulting in approximately 597,000 deaths, with Africa reporting 94% of the cases. In Brazil, the Amazon region is considered endemic, accounting for about 99% of cases. In 2023, more than 140,000 autochthonous cases (those acquired in the same location where they were diagnosed) were reported, along with 63 deaths, of which 17.3% were caused by *P. falciparum* and 82.7% by *P. vivax* and other species (Ministério da Saúde, n.d.(e)).

The malaria transmission cycle involves two hosts. During a blood meal, an infected female *Anopheles* mosquito inoculates sporozoite forms into the host. These parasites infect liver cells and transform into schizonts, which rupture and release merozoite forms. After the initial replication in the liver, the parasites undergo asexual multiplication in erythrocytes, where merozoites infect red blood cells. Ring-stage trophozoites then mature into schizonts, which rupture and release merozoites back into the bloodstream to infect new red blood cells. It is this rupture that leads to the clinical manifestations of malaria. Some parasites differentiate into male (microgametocytes) and female (macrogametocytes) gametocytes, which are ingested by the *Anopheles* mosquito during a blood meal from an individual with parasitemia. In the mosquito, parasite multiplication occurs through the sporogonic cycle, in which male gametes penetrate female gametes to form zygotes. These zygotes become motile and elongated, known as ookinetes, which invade the mosquito gut wall and develop into oocysts. The oocysts grow, rupture, and release sporozoites, which migrate to the mosquito's salivary glands and are inoculated into a new host (Tuteja, 2007; García-Basteiro et al., 2012).

The most common symptoms of malaria are high fever, chills, shivering, sweating, and headache, which may occur cyclically. Less common symptoms include nausea, vomiting, fatigue, and loss of appetite. Severe malaria is characterized by one or more of the following symptoms: prostration, convulsions, altered consciousness, hypotension, dyspnea or hyperventilation, and hemorrhages (Ministério da Saúde, n.d.(d)). It is noteworthy that individuals who have experienced multiple malaria episodes or are continuously exposed may develop naturally acquired immunity, providing protection against clinical symptoms and resulting in low parasitemia (Doolan et al., 2009).

Malaria diagnosis is performed through the visualization of the parasite in parasitological examinations, with the thick blood smear method considered the gold standard, although it can also be assessed using peripheral blood smears (Makler et al., 1998; O'Meara et al., 2006). Rapid tests detecting *Plasmodium* antigens are currently available, and in some cases, PCR may also be performed (Opoku Afriyie et al., 2023; de Fátima Ferreira-da-Cruz et al., 2025; Andrade et al., 2010).

Therapeutic strategies for malaria involve the use of artemisinin-based drugs or combination therapies, such as (1) artesunate and amodiaquine, (2) artemether and lumefantrine, (3) dihydroartemisinin and piperaquine, (4) artesunate and sulfadoxine–pyrimethamine, (5) artesunate and pyronaridine (Okombo and Fidock, 2025). The therapeutic combination reduces the risk of treatment failure, enhances parasite clearance, and improves treatment tolerance (Sudhakaran, 2025). In addition to therapeutic combinations, other drugs have been identified as promising candidates, including cabamiquine (a quinoline-carboxamide), cipargamin (a spiroindolone), and ganaplacide (an imidazolopiperazine) (Okombo and Fidock, 2025).

Moreover, vaccine development strategies are being explored as preventive measures (Naghizadeh et al., 2025). The transmission-blocking malaria vaccine (ProC6C-AlOH/Matrix-M™) was developed by incorporating three parasite proteins (Pfs230-Pro, Pfs48/45-6C, and CSP) and formulating them with two adjuvant combinations: aluminum hydroxide alone (ProC6C-AlOH) and aluminum hydroxide combined with the saponin-based Matrix-M™ adjuvant (ProC6C-AlOH/Matrix-M™) (Naghizadeh et al., 2025). Currently in Phase 1 clinical trials, this vaccine aims to prevent infection across multiple stages of the parasite's life cycle and has been shown to be safe, with the potential to induce high levels of specific IgG (Naghizadeh et al., 2025). Therefore, the development of new therapeutic and vaccine strategies is essential for controlling and reducing the transmission of the disease.

## 3 Principles and fundaments of diagnosis tests

Ideally, diagnostic tests should be able to correctly distinguish individuals who have the disease from those who do not, without necessarily assuming that the latter are healthy, as they may be affected by other conditions (Momeni et al., 2018). For this reason, in addition to understanding how diagnostic methods work and their respective advantages and limitations, it is essential to grasp the general concepts underlying diagnosis for the proper selection and interpretation of the tests performed. Factors such as sensitivity, specificity, predictive value, and reproducibility are crucial for choosing the most appropriate test to be used.

**Figure 2:**
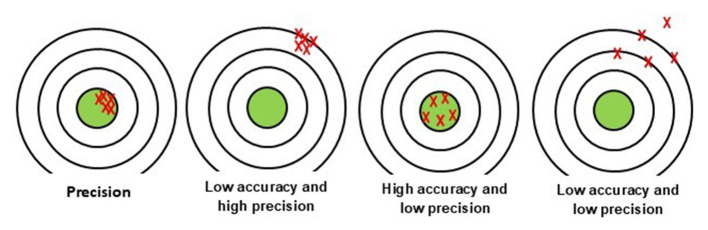
Schematic representation of accuracy and precision. Shots near the center indicate high accuracy; tightly grouped shots indicate high precision. Different combinations illustrate how a test can be precise but inaccurate, accurate but imprecise, both, or neither.

In this context, precision and accuracy may appear to be synonymous; however, they represent distinct measures. Accuracy refers to the closeness of the obtained result to the expected result, which corresponds to the “gold standard.” Mathematically, it can be defined as the proportion of true positives and true negatives over the total number of patients tested in the study, that is, true positives, true negatives, false positives, and false negatives. On the other hand, precision refers to the consistency of these results when repeated. In other words, if the test is repeated multiple times, the result should remain constant (Figure 2) (Momeni et al., 2018).

The parameters of precision include repeatability and reproducibility. Repeatability refers to the variation observed in tests performed using the same method, within the same laboratory, by the same operator, and employing the same equipment and materials over a short period of time. This reflects “intra-laboratory” consistency. Reproducibility, in contrast, refers to the variation in test results obtained with the same method but conducted across different laboratories, using different equipment, and by different operators. The concept of robustness is also closely related to precision, repeatability, and reproducibility, as it assesses the ability of a test to remain stable when subjected to minor variations. A test lacking robustness displays fluctuations in precision under such conditions, and therefore must be carried out with greater caution to avoid any unintended alterations (Caraguel et al., 2009).

The concept of sensitivity in a diagnostic test refers to its ability to correctly identify individuals who truly have the disease/infection. In other words, it is the capacity of the test to yield a “positive result” when the patient is indeed positive. Another important statistical concept is specificity, which considers the complementary aspect, assessing whether the test can correctly identify individuals who do not have the disease. In other words, it is the ability of the test to produce a “negative result” when the patient is truly negative (Wong and Lim, 2011).

Two other parameters used in the evaluation of diagnostic tests are the positive predictive value (PPV) and the negative predictive value (NPV), which are related to disease prevalence (π), unlike the previously mentioned parameters. The PPV represents the probability that a positive test result is a true positive, while the NPV represents the probability that a negative test result is a true negative (Wong and Lim, 2011).

Diagnostic methods can be divided into two main groups: direct and indirect methods. Direct diagnostic methods aim to detect components of the pathogen, that is, to observe its presence directly. These methods are particularly important for early diagnosis when the pathogen load is low. Indirect methods, on the other hand, involve the detection of evidence indicating the presence of the parasite, such as the production of antibodies against the pathogen or specific cytokines. Although they rely on the host's immune response and are not the preferred method for detecting early infection, indirect methods can provide crucial support for epidemiological monitoring, allowing the detection of not only active disease but also prior exposure to pathogens (Nascimento et al., 2024). Moreover, certain methods, such as ELISA assay and Western-Blotting, can accommodate both approaches depending on the intended objective. These techniques can be designed either to detect antigens of the parasite itself or to identify antibodies that indicate its presence.

Assay performance can be affected not only by technical handling and sampling issues but also by biological factors that alter parasite load, immune responses, antigen or antibody levels (Zakhour et al., 2023; Martiáñez-Vendrell et al., 2022). Coinfected patients often show overlapping symptoms that delay correct testing, and interactions between parasites may exacerbate or suppress immune pathways, hindering detection (Ornellas-Garcia et al., 2023). In coinfections involving immunosuppressive settings such as HIV, up to half of Leishmania–HIV patients lack detectable antibodies, reducing serological sensitivity (Harms and Feldmeier, 2002). While microscopy is usually unaffected, serological and molecular tests can be compromised by coinfection.

To address these diagnostic challenges and account for different stages of infection, more than one test may be required for confirmation. For Chagas disease, for example, microscopy is typically restricted to the acute phase, whereas chronic infection, characterized by low parasitemia, relies on serological assays (Ministério Da Saúde, 2018).

## 4 Rapid tests for the diagnosis of parasitic diseases

Rapid tests for the diagnosis of parasitic diseases are qualitative, easy to perform, and can be used in the field and in rural areas (Shah et al., 2014). They are recommended by the WHO as they enable early and rapid diagnosis in a few minutes and are also used in screening pregnant women to prevent congenital infections (Shah et al., 2014; World Health Organization, 2025b).

Immunochromatographic tests are rapid tests widely used in the diagnosis of diseases such as malaria, Chagas disease, and leishmaniasis, among others (World Health Organization, 2025b). These assays are generally based on the lateral flow principle, in which antigens or antibodies present in the sample interact with labeled conjugates (by dye or particle, such as colloidal gold) and are visualized in reactive lines on a nitrocellulose membrane (Pedreira-Rincón et al., 2025). The device is assembled in a cassette format, in which the sample is applied followed by a buffer solution, which promotes migration by capillarity to the test (T) and control (C) regions, ensuring the validity of the test (World Health Organization, 2025b). When positive, the rapid test shows a line in the test region (T), which results from the interaction between the antigen and the specific antibody; in addition, there is always a control line (C) that must be marked in a valid test (Pedreira-Rincón et al., 2025).

For malaria diagnosis, rapid diagnostic tests allow the differentiation between *P. falciparum*, non-*Pf* infections, and mixed infections. The OptiMAL^®^ test has high sensitivity (>90%) and contains the monoclonal anti-malaria pLDH (plasmodial lactate dehydrogenase) antibody, which detects antigens from *P. vivax, P. ovale, and P. malariae*. However, it is important to mention that deletions in hrp2 and hrp3 can reduce sensitivity of tests such as OptiMAL and Bioline since these deletions can lead to false-negative results in rapid diagnostic tests (Pereira Filho, 2023). The diagnosis is made within 20 to 60 minutes using whole blood samples (Valéa et al., 2009).

A positive result for *P. falciparum* is indicated by a band in the P.f region caused by the recognition of HRP, *P. falciparum* antigen, and a band in the Pan region (Ministério da Saúde, 2005a). For the species *P. vivax, P. ovale*, and *P. malariae*, a positive diagnosis will generate an isolated band in the Pan region, caused by the recognition of pLDH (Palmer et al., 1998). If the control band is not marked, the test is considered invalid and must be repeated (World Health Organization, 2023b). The Bioline™ Malaria Ag P.f/P.v test also contains pLDH with lactate dehydrogenase, which is more specific for the identification of *P. vivax* antigen (Madamet et al., 2024).

The ParaSight test is also an alternative rapid diagnostic test for malaria, being specific for *Plasmodium falciparum*. This test detects the parasite's Histidine-Rich Protein II (HRP-II) in blood using dye-labeled antibodies for visual readout. It is suitable for settings without microscopy, with good sensitivity and specificity, and can also be part of a more advanced computer vision platform developed by Sight Diagnostics for automated malaria diagnosis (Pieroni et al., 1998).

Another test used for malaria diagnosis is the OnSite^®^ malaria Pf/Pv Ab. This test consists of a Pf line pre-coated with recombinant Pf MSP antigen for the detection of antibodies to *P. falciparum* and a Pv line pre-coated with Pv MSP antigen for the detection of antibodies to *P. vivax* (Instruções de Uso, 2020).

It is important to note that the WHO has reported 38 countries with strains of *P. falciparum* with deleted genes for HRP 3 and HRP 2, including Brazil, which may cause false negative results for the detection of *P. falciparum* in these tests (Table 1) (World Health Organization, 2025b; Agaba et al., 2024).

**Table 1:** Parameters of rapid tests for the diagnosis of malaria.

**Tests**	**Susceptible species**	**Sensitivity**	**Specificity**	**Methodology**	**References**
OnSite Malaria Pf/Pan Ag^®^	*P. falciparum P. vivax*	94.2% for *P. falciparum* and 97.3% for *P. vivax* (comparative analysis of 372 blood samples)	99.5% for *P. falciparum*100% and 98.7% for *P. vivax* (comparative analysis of 372 blood samples)	Indirect Direct immunochromatographic Detects pLDH e HRP from HRP 2 antigen from *P. falciparum* and LDH antigen from *P. vivax, P. ovale*, and *P. malariaevivax*.	Mohon et al., 2012
ParaSight^TM^-F	*P. falciparum*	91.6% (comparative analysis of 520 patient blood samples)	99.4% (comparative analysis of 520 patient blood samples)	Direct immunochromatographic Detects HRP II antigen from *P. falciparum*	Banchongaksorn et al., 1996
OptiMAL-IT Malaria Pf/Pan Ag^®^	*P. falciparum P. vivax P. ovale P. malariae*	98.7% (comparative analysis of 464 patient blood samples)	96.2% (comparative analysis of 464 patient blood samples)	Direct immunochromatographic Detects LDH HRP 2 antigen from *P. falciparum* and LDH, antigen from *P. vivax, P. ovale*, and *P. malariae*	Ditombi et al., 2020
BIOLINE™ Malaria Ag P.F./P.V	*P. falciparum P. vivax*	95.3% (comparative analysis of 229 patient blood samples)	100% (comparative analysis of 229 patient blood samples)	Direct immunochromatographic Detects *P. falciparum* HRP 2 antigen and pLDH antigen for *P. vivax*.	Madamet et al., 2024

Chagas disease is diagnosed using rapid tests, such as the OnSite^®^ Chagas Ab Combo Rapid Test, TR Chagas BioManguinhos (Fiocruz/Brazil), Chagas Detect™ Plus (InBios, USA), SD Bioline™ Chagas (Abbott/Alere), among others (Egüez et al., 2017). These tests are immunochromatographic and detect antibodies to *T. cruzi* antigens as B13, IF8, and H49/JL7, but the sensitivity and specificity differ between each test (Table 2) (Egüez et al., 2017; Ji et al., 2009).

**Table 2:** Parameters of rapid tests for the diagnosis of Chagas disease.

**Tests**	**Sensitivity**	**Specificity**	**Methodology**	**References**
TR Chagas BioManguinhos	100% [serum samples from patients (*n* = 1,000,000)]	100% [serum samples from patients *n* = 1,000,000]	Indirect immunochromatographic Detects anti-T*.cruzi*	Santos et al., 2024
On Site^®^ Chagas Ab CTK Biotech	97.9% (comparative analysis of 320 patient serum)	98.8% (comparative analysis of 320 patient serum)	Indirect immunochromatographic Detects anti-T. *cruzi* IgG	Ji et al., 2009
Chagas Detect™ Plus	96.2% (the analysis was performed on paired whole blood and serum samples from 385 individuals)	>95% (the analysis was performed on paired whole blood and serum samples from 385 individuals)	Indirect immunochromatographic Detects anti-*T. cruzi* IgG	Shah et al., 2014
SD BIOLINE™ Chagas	99.3% (comparative analysis of 320 patient serum)	100% (comparative analysis of 320 patient serum)	Indirect immunochromatographic Detects anti-T*. cruzi*	Ji et al., 2009

The Chagas TR BioManguinhos rapid test is made in Brazil at BioManguinhos, part of the Fundação Oswaldo Cruz (Fiocruz), and is one of the tests used to diagnose Chagas disease (Iturra et al., 2023). Its methodology is based on a lateral-flow immunochromatographic assay using an indirect immunoassay format, and in Brazil it has demonstrated excellent performance, with sensitivity and specificity approaching 100% in multicenter evaluations (Santos et al., 2024). It uses whole blood, serum, or plasma samples and has two strips, one of which consists of a membrane A, pre-coated with *T. cruzi* antigen, detecting anti-*T. cruzi* antibodies (Iturra et al., 2023).

When the result is positive in any of the rapid tests for Chagas disease, a serological test, such as ELISA, is also indicated to confirm the diagnosis (Shah et al., 2014; Egüez et al., 2017; Iturra et al., 2023).

The TR-DPP^®^ Canine Visceral Leishmaniasis (CVL) is a rapid test produced by BioManguinhos/Fiocruz for the diagnosis of CVL. The test shows high overall specificity (96%); however, sensitivity varies according to the dogs' clinical status, being higher in symptomatic dogs (98%) than in asymptomatic dogs (47%) (Table 3) (Grimaldi et al., 2012). It is performed using the indirect immunochromatographic technique to detect antibodies present in blood, plasma, or serum samples against recombinant proteins of *Leishmania infantum* rK28, a chimeric protein formed by fragments of rK9, rK26, and rK39 located on the nitrocellulose membrane. In addition to the DPP test, SensPERT^®^ test (Dechra^®^, Paraná, Brazil) and Alere^®^Leishmaniasis Ac test are also used as diagnostic rapid test (Table 3) (Grimaldi et al., 2012; Morgado et al., 2025; Pereira et al., 2024).

**Table 3:** Parameters of rapid tests for the diagnosis of canine visceral leishmaniasis.

**Tests**	**Sensitivity**	**Specificity**	**Methodology**	**References**
TR-DPP^®^ BioManguinhos LVC	98% [analyzed using serum samples with negative diagnostic results (*n* = 59) and cross-reaction control sera (*n* = 11) from animals]	96% [analyzed using serum samples with negative diagnostic results (*n* = 59) and cross-reaction control sera (*n* = 11) from animals]	Indirect immunochromatographic Detects antibodies against recombinant protein rK28 (K9, rK26, and rK39) from *Leishmania infantum*	Grimaldi et al., 2012
SensPERT^®^ test (Dechra^®^, Paraná, Brazil)	96.5% (comparative analysis of 30 canine serum samples)	83.8% (comparative analysis of 30 canine serum samples)	Indirect immunochromatographic Detects antibodies against recombinant protein (rK29 and rK39) from *Leishmania infantum*	Pereira et al., 2024
Alere^®^ Leishmaniasis Ac Test (Alere, São Paulo, SP, Brazil)	93.1% (comparative analysis of 30 canine serum samples)	100% (comparative analysis of 30 canine serum samples)	Indirect immunochromatographic Detects antibodies against recombinant protein (rK28) from *Leishmania infantum*	Pereira et al., 2024

The rK39 ICT Kalazar Detect™ (InBios International) test is used for the diagnosis of visceral leishmaniasis in humans and is based on the rK39 protein, a 39-amino acid protein encoded by a gene related to kinesin in *Leishmania infantum* amastigotes (Bezuneh et al., 2014). The test detects antibodies against *Leishmania* rK39 in serum samples (Bezuneh et al., 2014). The detection of antibodies against rK39 has a sensitivity. The test has 87.5% sensitivity in serum samples, 96.4% in urine samples and 90.6% in saliva samples (Table 4) (Bezuneh et al., 2014; Tamir et al., 2024; Vaish et al., 2012).

**Table 4:** Parameters of rapid tests for the diagnosis of human visceral leishmaniasis.

**Test**	**Sensitivity**	**Specificity**	**Methodology**	**Reference**
Kalazar Detect™ rapid test for visceral leishmaniasis	87.5% (comparative analysis of 63 patient serum)	>90% (comparative analysis of 206 patient serum)	Indirect immunochromatographic Detects antibodies against recombinant rK39 protein *L. infantum*	Bezuneh et al., 2014

## 5 Diagnosis based on antigen-antibody interaction

Antigen-antibody interaction refers to the specific chemical binding between antibodies, produced by B lymphocytes, and antigens during immune responses. This reaction is a fundamental mechanism by which the body defends itself against complex foreign molecules, including pathogens and their associated toxins. In the bloodstream, an antigen-antibody complex forms when a highly specific antigen binds to its corresponding antibody. The resulting immune complex is then transported to cellular systems, where it can be neutralized or eliminated. In the body, these interactions support antibody-mediated immunity against infectious diseases and may also contribute to tissue damage in hypersensitivity or autoimmune conditions.

In laboratory settings, antigen-antibody interactions are widely employed for diagnosing infections in epidemiological studies and for identifying both infectious and non-infectious agents, such as enzymes. These reactions allow for the detection and quantification of either antigens or antibodies. When performed *in vitro*, they are referred to as serological reactions. The binding between antigen and antibody is a reversible bimolecular association that does not induce permanent chemical changes in either component (Mir, 2020).

### 5.1 Enzyme-linked immunosorbent assay (ELISA)

ELISA is an immunological technique widely used to detect the presence of proteins, antibodies, hormones, and other molecules in a sample. It is based on the highly specific and sensitive interaction between antigens and antibodies, mediated by enzymatic reactions (Pereira Filho, 2023). The assay involves the incubation of reagents, and its final outcome is the development of a measurable color change, which can be quantified using a spectrophotometer. This quantification enables accurate interpretation of the results. As a diagnostic method, ELISA aims to assess specific concentrations of antigens or antibodies in order to determine whether the analyzed serum can show the infection and to evaluate the level of immune protection (Crowther, 2008). Moreover, due to its high reproducibility and ability to generate a large number of results within a single experiment, this technique is widely employed in both scientific research laboratories and clinical diagnostic settings (Hosseini et al., 2017).

**Figure 3:**
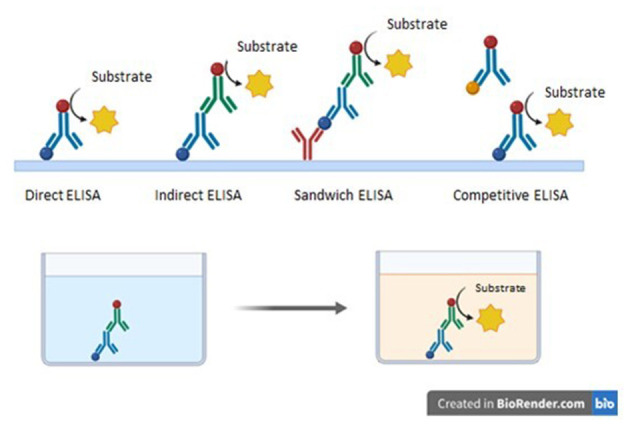
Formats of ELISA assay. The yellow star represents the substrate, the blue sphere represents the antigen, and the red sphere represents the enzyme. Direct ELISA: Detects antibodies by binding a single enzyme-conjugated antibody directly to the immobilized antigen. Fast and simple, but less sensitive. Indirect ELISA: Uses a primary antibody against the antigen and a secondary enzyme-conjugated antibody for detection, increasing sensitivity but with higher risk of cross-reactivity. Sandwich ELISA: Employs two specific antibodies binding to different epitopes of the same antigen, providing high specificity and sensitivity. Requires the availability of two compatible antibodies. Competitive ELISA: Based on competition between sample antigen and labeled antigen for antibody binding. Useful for small or low-immunogenic antigens, but usually less sensitive than sandwich ELISA. Image: Created using BioRender.

The ELISA assay can be either qualitative or quantitative, and in general, the assay can be categorized into four main formats: direct ELISA, indirect ELISA, sandwich ELISA, and competitive ELISA (Figure 3). Direct ELISA is the most practical form of the assay and aims to verify the presence or even quantify the amount of antibody, allowing the identification of whether an individual or animal has been exposed to a given pathogen. In this assay, antigens derived from the sample of interest are immobilized, followed by the addition of a primary antibody conjugated to an enzyme (peroxidase or alkaline phosphatase), which enables the reaction and color change upon contact with the substrate due to enzymatic catalysis. The intensity of the color is visually estimated and is proportional to the concentration of the antibody under investigation. Since only one antibody is added, there is no possibility of cross-reactivity in this type of assay. Furthermore, it can be performed in a shorter time compared to other formats (Lin, 2015). On the other hand, the assay has disadvantages such as low sensitivity, due to the use of only one antibody, and consequently reduced signal amplification (Crowther, 2008; Gan and Patel, 2013).

The indirect ELISA involves the addition of the antigen for plate sensitization, followed by the addition of the sample that may contain the antibody of interest. Afterwards, a second antibody (secondary antibody conjugated to an enzyme), which is specific to the primary antibody, is added. Finally, the substrate is introduced, and the enzyme generates the detection signal resulting from the chemical reaction: enzyme, hydrogen peroxide, and substrate. The advantages of Indirect ELISA include versatility, as a single primary antibody can be used to detect different antigens; and sensitivity, as it is generally more sensitive than direct ELISA by allowing detection of multiple secondary antibodies bound to a single primary antibody, amplifying the detection signal (Lin, 2015; Tabatabaei and Ahmed, 2022). However, signal amplification may also increase the likelihood of cross-reactivity, thereby raising background signal levels (Crowther, 2008).

The sandwich ELISA is widely used to detect specific antigens at low concentrations. In this method, two different specific antibodies are employed: a capture antibody and a detection antibody. The capture antibody is immobilized on the plate, after which the sample is added. The antigen present binds to the capture antibody, followed by the addition of the detection antibody conjugated to an enzyme, thus forming an antibody-antigen–antibody complex. After washing, the substrate is added, and the enzyme produces a colorimetric detection signal. Sandwich ELISA has advantages such as high specificity, since two different antibodies bind to distinct epitopes of the antigen, enabling more specific detection and minimizing interference and cross-reactivity. It is often considered more sensitive than Direct or Indirect ELISA (Pereira Filho, 2023). The disadvantages include the requirement for two specific antibodies that must not compete for the same epitope, which can be challenging and costly, as well as the limitation that this assay cannot be applied to antigens with only one epitope, since they do not allow simultaneous binding of two antibodies (Crowther, 2008; Gan and Patel, 2013).

In the competitive ELISA, the antigen is first incubated with a specific antibody conjugated to an enzyme. This antigen–antibody complex is then added to a plate pre-coated with a capture antibody. The labeled antigen competes with the antigen from the sample for binding to the capture antibody on the plate surface. The higher the amount of antigen in the sample, the less labeled antigen will bind to the capture antibody. In this case, the enzymatic activity is measured as inversely proportional to the amount of antigen present in the sample. Although Competitive ELISA is less common than other formats, it presents specific advantages such as broad applicability, being suitable for the detection of small, poorly immunogenic antigens where other ELISA formats may not be appropriate, and high specificity due to the competition principle (it can be highly sensitive for detecting specific antigens) (Pereira Filho, 2023; Lequin, 2005). However, if there are substances in the sample capable of nonspecific competition, this may compromise the success of the technique. In addition, it is considered less sensitive than Sandwich ELISA, precisely due to the competition process, which naturally results in a lower signal (Lequin, 2005).

The selection of the most appropriate format depends directly on the specific experimental question being addressed. Each variation offers the possibility of optimizing aspects of the test, such as enhancing sensitivity, diversifying the types of antibodies employed, and amplifying the detection signal. Consequently, ELISA stands out as a highly versatile technique, applicable across a wide range of research and diagnostic contexts (Pereira Filho, 2023).

The execution of the ELISA test follows a sequence of essential steps. Initially, sensitization is performed, during which the antigen or antibody is adsorbed onto the surface of the plate. The analysis is typically conducted in a microtiter plate specifically coated for the assay of interest, where specific antigens or antibodies are immobilized on the well surfaces, creating a “coating” capable of capturing the target. These plates are usually made of polystyrene, a material with a protein-adsorbing surface, and feature either flat- or U-shaped bottoms (Engvall et al., 1971). The type of ELISA being performed determines which antigens or antibodies are required at this stage, as well as their concentrations and amounts.

The next step involves blocking the free binding sites to prevent nonspecific interactions. For this purpose, bovine serum albumin (BSA) is commonly used, as it adheres to the plate and prevents unwanted binding of reagents that could otherwise generate false results (Lequin, 2005). After blocking, the plate is washed with PBS containing Tween 20 to remove excess blocking agent. Between each step, careful washing is required to eliminate unbound reagents from the supernatant, preventing interference with the assay outcome. Washing is performed with a buffer solution supplemented with a nonionic detergent, typically phosphate-buffered saline (PBS), prepared by diluting a 10 × stock solution to 1 × , in combination with Tween 20 (Crowther, 2008). The number of washes depends on the specific protocol being followed, and they may be performed using an automated plate washer or manually with pipettes and other tools.

Subsequently, the samples can be incubated, followed by another wash with PBS + Tween 20 to remove unbound material. A secondary antibody conjugated to an enzyme is then added, followed by additional washes to eliminate unbound antibodies. To generate a colorimetric signal at the end of the experiment, the primary antibody (in direct ELISA) or the secondary antibody (in indirect, sandwich, or competitive ELISA) is conjugated to an enzyme, most commonly horseradish peroxidase (HRP) or alkaline phosphatase (AP) (Crowther, 2008).

Finally, a chromogenic substrate is added, which reacts with the enzyme to produce a colorimetric signal. Depending on the enzyme–substrate combination, the final product may exhibit a blue or orange color (Pereira Filho, 2023). The most frequently used substrates are o-phenylenediamine dihydrochloride (OPD) and 3,3′,5,5′-tetramethylbenzidine (TMB) (Crowther, 2008). To allow proper measurement, the enzymatic reaction must be stopped before the plate is read in an ELISA plate reader. This is achieved by adding an acid, most commonly sulfuric acid (H_2_SO_4_) (Crowther, 2008) or hydrochloric acid (HCl) (Gan and Patel, 2013). After stopping the reaction, the plate must be read in an ELISA reader within 30 min. The wavelength selected for measurement depends on the stop solution employed in the assay.

In pathogen diagnosis, ELISA is an indispensable technique, being employed from the confirmation of protozoan presence to the quantification of its peptides, particularly in relation to the most common protozoal infections in Brazil. Numerous examples could be cited regarding the role of ELISA in diagnosing these diseases. In leishmaniasis, ELISA can be applied in patient screening (Bia et al., 2025), in diagnostic confirmation (Casas et al., 2024), after chromatography (Pradella et al., 2025), and in the quantification of molecules such as cytokines following disease resolution (Jawalagatti et al., 2023). In Chagas disease, ELISA can be used in a variety of ways, from the investigation of antigens or antibodies (Suescún-Carrero et al., 2022) to direct disease diagnosis, being the most widely employed serological technique (Ossowski et al., 2024). In malaria, ELISA plays multiple roles, from the detection of antigens in the human host or in the vector, to the identification of specific antibodies in the human host (Hendershot et al., 2021; Jang et al., 2022), and even in epidemiological studies to assess population exposure to the parasite (Ventocilla et al., 2024).

Over the years, the continuous evolution and refinement of this technique has broadened its range of applications, enhancing its sensitivity, specificity, and reliability, assisting in improving the technique in order to avoid false positives or negatives. Despite some limitations, the benefits associated with the ELISA assay, such as ease of implementation, high reproducibility, and relatively low cost, significantly outweigh its disadvantages. For these reasons, ELISA remains one of the most important analytical tools employed in clinical and research laboratories worldwide (Pereira Filho, 2023).

### 5.2 Enzyme-linked immunospot (ELISPOT)

**Figure 4:**
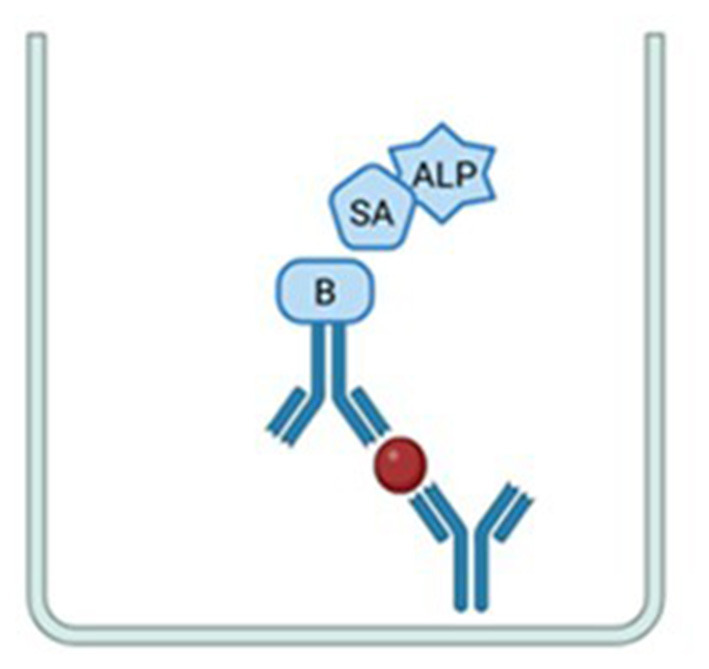
Representation of the immunochemical “sandwich” in the Elispot technique. The image shows the capture antibody bound to the plate membrane, the captured analyte represented by the small red dot, the detection antibody conjugated to biotin (B) bound to the streptavidin–alkaline phosphatase conjugate (SA and ALP). Image: Created using BioRender.

ELISpot assay is an immunoassay used to quantify analyte-secreting cells and these analytes may include cytokines, immunoglobulins, or other target proteins that are secreted upon specific cellular stimulation and subsequently captured by specific antibodies (Gertow, 2022). Since this technique employs enzymes conjugated to antibodies and results in the formation of precipitates in the form of small dots or “spots,” researchers named the assay ELISPOT, from the English enzyme-linked immunospot (Czerkinsky et al., 1983). Since then, the method has been refined to reach its current format, available as commercial kits or with separate reagents, in which, similar to the ELISA technique, consists of an immunochemical “sandwich” combining antigens with pairs of antibodies (capture and detection), as illustrated in Figure 4.

Despite this similarity, it is important to emphasize that these assays provide different types of information and can be used in a complementary manner: considering a given cytokine, for example, ELISA enables the measurement of the actual concentration of the molecule present in the supernatant, i.e., the total amount of cytokine secreted. In contrast, ELISPOT allows the determination of the frequency of the cells secreting that particular cytokine (Kalyuzhny, 2005). Because ELISPOT can detect the capacity of even a single cell to secrete cytokines, it is characterized as a much more sensitive technique—up to 100 times more sensitive than an ELISA assay—thereby enabling the detection of cytokines at levels previously undetectable in the supernatant (GEGINAT GpS, 2005).

**Figure 5:**
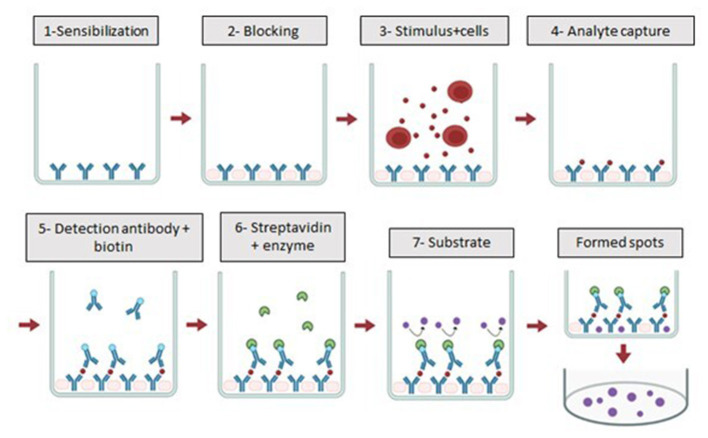
Stages of the ELISPOT technique. Schematic representation of the ELISpot assay. The detection antibody conjugated to biotin (blue circle) binds to the analyte captured by the capture antibody (red circle). Streptavidin–enzyme conjugate (green circle) is then added and, in the presence of substrate (purple circle), forms insoluble precipitates visualized as spots. Image: Produced using BioRender.

In general, the ELISPOT assay can be divided into different stages that take up to 3 days. Briefly, it begins with the coating phase, in which the plate is sensitized with the capture antibody. For this, within a biological safety cabinet, 96-well ELISPOT plates containing a polyvinylidene fluoride (PVDF) membrane are pretreated with ethanol, followed by the addition of the capture antibody and subsequent overnight incubation at 4–8 °C. This sensitization step is unnecessary when using pre-coated ELISPOT plates, a more expensive material, that allows one less day of experiment. On the second day the selected stimulus (e.g., synthetic peptides corresponding to epitopes of interest) is added first, followed by the cells (typically peripheral blood mononuclear cells, PBMCs). In a CO_2_ incubator (5% CO_2_, 37 °C), the stimulated cells secrete the analyte. This secretion period may range from 12 to 48 h, depending on the analyte of interest. On the third day, after cell removal, the detection antibody specific to the analyte of interest is added, followed by an enzyme conjugate and a chromogenic substrate for the enzyme, BCIP/NBT-plus. Upon reaction, this substrate is converted into a purple precipitate that deposits onto the PVDF membrane in the form of spots and the membrane must be washed with water and allowed to dry completely for at least 24 h before reading the plate in an ELISPOT reader. After drying, the plates can be stored for long periods, thus allowing the technique to be directly performed in endemic areas and the plates can be transferred for analysis at research centers (Ji and Forsthuber, 2016) (Figure 5).

Several studies in this field have shown that the frequency of IFN-γ-producing CD8+ T cells specific for *T. cruzi* is inversely correlated with the severity of chronic Chagas disease (Laucella et al., 2004). Similarly, research comparing children infected with *T. cruzi* at early stages to adults in the chronic phase demonstrated that the cellular immune response in children is characterized by polyfunctional T cells, with IFN-γ production up to five times higher than in adults (Albareda et al., 2013). ELISPOT assays have also shown that patients with high pre-treatment frequencies of IFN-γ- and IL-2-producing T cells who received benznidazole exhibited progressively lower antibody titers after treatment completion, as assessed by serology, the current standard for indicating cure through the absence of specific antibodies (Alvarez et al., 2016). These findings, together with other techniques, can contribute to a more precise diagnosis of Chagas disease. There are also studies reporting the use of the ELISPOT technique for the investigation of leishmaniasis, as in the study of the follow-up of two pregnant women with cutaneous leishmaniasis, in which ELISPOT assays were used. In this paper, it was observed that IFN-γ levels increased as lesions healed. This indicates alterations in the maternal immune system, affect susceptibility to *Leishmania braziliensis* and result in the worsening of the disease (Conceição-Silva et al., 2013). Although the technique was not used for diagnosis itself, it was associated with other techniques in the context of prognosis, a process closely linked to diagnosis and essential for the determination of disease progression. From the perspective of malaria, to date, studies described in the scientific literature focus mainly on aspects such as pathogenesis, epitope identification, and vaccine development (Ventocilla et al., 2024; Atre et al., 2019; John et al., 2004; Ganeshan et al., 2016). Despite the limited number of studies using ELISPOT as an ally in diagnosis, based on the studies cited, this technique may support broader applications, including disease monitoring, assessment of treatment efficacy, and determination of patient cure, as already demonstrated in various tropical diseases (Lima-Junior et al., 2017).

### 5.3 Immunohistochemistry

Direct parasitological tests, including histopathology, immunohistochemistry, cytology, and immunocytochemistry, are considered the gold standard for the diagnosis of parasitic diseases. Similar to histopathology, immunohistochemistry requires tissue collected by biopsy, necessitating trained professionals to perform the procedure (Conceição-Silva, 2024). Thus, the Immunohistochemistry (IHC) is a technique that employs the antigen–antibody reaction to detect target molecules in tissue sections. It allows the *in situ* identification of various structures that may be associated with different diseases and is widely used in anatomical pathology (Ferro, 2014). In the context of diagnosing parasitic diseases, IHC is used to detect antigens of the specific parasite targeted by the test, offering greater specificity compared to conventional histopathology and allowing the differentiation of morphologically similar parasites (Ezyaguirre et al., 2011; Conceição-Silva, 2024). It is very useful for identifying parasites in low amounts that may be difficult to detect using routine staining techniques (Ezyaguirre et al., 2011). The IHC technique can be performed using different methods to identify a specific antigen. However, IHC is considered a complex technique, and several factors can affect the quality of staining. These factors range from early steps, such as tissue fixation and antigen retrieval, to later steps, such as the choice of detection method (Nielsen, 2021). In the direct method, the primary antibody conjugated to a label bind directly to the target antigen, eliminating the need for a secondary antibody. In the indirect method, a primary antibody detects the target antigen, followed by a secondary antibody raised against the immunoglobulin of the species in which the primary antibody was produced. The secondary antibody must be conjugated to a substance that enables visualization of the antigen–antibody complex (Ferro, 2014). In addition to direct and indirect methods, other methods can also be employed, including: Peroxidase Anti-Peroxidase (PAP) method, Alkaline Phosphatase Anti-Alkaline Phosphatase (APAAP) method, avidin-biotin methods, and polymer methods (Murphy et al., 1997).

In the initial steps of the technique, the sample preparation and tissue fixation, are crucial for the preservation, stabilization, and protection of samples to be used in subsequent analyses (Ferro, 2014). Two types of fixations can be employed for IHC: the tissue can be cryopreserved at low temperatures, embedded in an appropriate resin, or fixed in formaldehyde and subsequently embedded in paraffin (Conceição-Silva, 2024). Cryopreservation offers the advantage of a faster IHC procedure compared to paraffin-embedded material. However, the freezing process can cause tissue damage, affecting tissue integrity and, consequently, IHC performance (Colley and Ronald, 2021). The most commonly used fixative is 10% neutral-buffered formalin, which penetrates tissues effectively and preserves morphological details for subsequent paraffin embedding. However, antigens can be masked by formaldehyde, which causes conformational changes in epitopes, resulting in the loss of the antibody's ability to interact with the target antigen, requiring antigen retrieval (Shi and Taylor, 2021).

The antigen retrieval involves treating the tissue to restore the structure of the protein that has been masked by formaldehyde (Ferro, 2014). This can be achieved through enzymatic digestion or heat-induced methods (Shi and Taylor, 2021).

This process can reverse structural modifications induced by formalin, provided that the primary protein structure, determined by the amino acid sequence, remains intact (Ferro, 2014). Optimal conditions for antigen retrieval depend on the antibody to be used. Therefore, when selecting an antibody, it is essential to verify its suitability for paraffin-embedded material and determine the most appropriate retrieval method.

**Figure 6:**
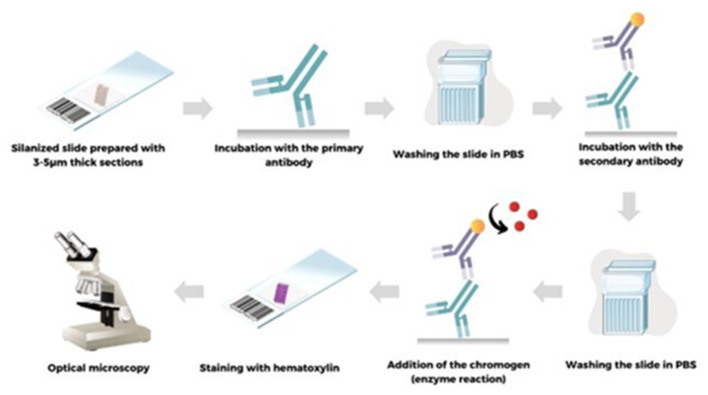
Schematic representation of the main steps of immunohistochemistry.

The antibody selection depends on the laboratory, considering factors from tissue processing and fixation to tissue species. Correct dilutions prepared consistently ensure high specificity with minimal background. The dilution, incubation time, and temperature of the primary antibody are also crucial steps. There is an inverse relationship between antibody concentration and incubation time: higher concentrations require shorter incubation (Jensen, 2021). Regarding detection methods, in the case of an antibody conjugated to an enzyme (HRP), a chromogen (DAB or AEC) must be added for the development of the enzymatic reaction. The oxidation by Peroxide (HRP) results in a brown (DAB) or red (AEC) precipitate, indicating the parasite localization in the tissue (Jensen, 2021). Finally, many counterstains can be used in IHC, with hematoxylin being the most common. Nuclear staining in blue improves visualization of tissue morphology and provides contrast with the brown DAB or red AEC staining (Jacobsen et al., 2021). The Figure 6 provides a summary representation of the IHC procedure.

In *Leishmania* infection, immunohistochemistry is considered a reference test for parasitological diagnosis, allowing correlation of the parasite with tissue lesions and the detection of active infection (Conceição-Silva, 2024). In the case of chronic granulomatous leishmaniasis with a small number of parasites in the sample, immunohistochemical staining has been a very useful diagnostic technique for parasite identification (Ezyaguirre et al., 2011). However, although it is a more sensitive technique in relation to histopathology, there may be cross-reactions with other pathogens such as Trypanosoma cruzi, requiring additional tests for an accurate diagnosis (Ferreira et al., 2022). These cross-reactions may be caused by the use of polyclonal antibodies or hyperimmune sera processed in-house, which can produce nonspecific bindings, considered one of the limitations of the technique (Conceição-Silva, 2024).

### 5.4 Flow cytometry

Flow cytometry is a methodology capable of simultaneously measuring, within a short period of time, multiple physical and biological characteristics of a single cell or particle, such as size, granularity, morphology, and function, as the cell flows in suspension (Adan et al., 2017). This measurement is achieved through the detection of fluorescence emission from fluorescent molecules—known as fluorochromes—in samples previously prepared and labeled with monoclonal antibodies conjugated to fluorochromes of interest (Ferraz and Bertho, 2013). Due to its ability to analyze several parameters simultaneously at the single-cell level, flow cytometry has become an essential technique for multiparametric analyses, and is widely employed in the immunophenotyping of peripheral blood cells, apoptosis assays, hematology, immunology, cytokine detection, immunogenetics, among others (dos Santos et al., 2017).

The basic principle of flow cytometry relies on the passage of labeled cells in suspension through a laser beam that excites the fluorochrome(s), which in turn emit light at specific wavelengths (colors) captured by strategically positioned detectors. The fluorochromes are typically conjugated to monoclonal antibodies used to label cells, with each fluorochrome exhibiting a distinct excitation and emission spectrum (Flores-Montero et al., 2019; Bertho et al., 2024; Actor, 2024). These conjugated antibodies specifically interact with molecular targets located on the cell surface or within the cytoplasm. Such targets are known as clusters of differentiation (CDs) (Bertho et al., 2024). Thus, combining multiple anti-CD antibodies conjugated to different fluorochromes enables the simultaneous identification of diverse cell populations and their functional states within a single sample in a relatively short time frame.

The detectors, photomultiplier tubes (PMTs), measure the light absorbed or scattered by the sample and convert it into electronic signals that are processed by a computer, enabling morphological and functional analyses using dedicated multiparametric software. The results are commonly displayed as fluorescence intensity plots, depending on the fluorochrome(s) employed, providing information on cellular morphology such as size and granularity (Bajgelman, 2019).

An essential step in flow cytometry is the preparation of biological samples, such as peripheral blood, cell cultures, cerebrospinal fluid, tissue, and bone marrow, among others. To allow proper evaluation, cell suspensions are required; therefore, sample dissociation is necessary to prevent the formation of cellular aggregates that may damage the instrument and generate unreliable results (Ferraz and Bertho, 2013). Additional procedures may be required during sample preparation, including the use of trypsin to detach adherent cells, removal of unwanted contaminating material, or lysis of red blood cells in peripheral blood (Bio Rad Antibodies, n.d.). Subsequently, molecular targets to be labeled with monoclonal antibodies must be selected. The choice of fluorochromes depends on the optical configuration of the flow cytometer (Ferraz and Bertho, 2013).

One of the most commonly applied protocols in flow cytometry is the labeling of cell surface proteins, as the plasma membrane is readily accessible to antibodies (Adan et al., 2017). Intracellular proteins can also be detected by flow cytometry; however, this requires that cells be treated with protein transport inhibitors to ensure retention of secreted proteins. The cells are then fixed, typically with formaldehyde, and permeabilized using detergents or alcohol, allowing antibody penetration into the cytoplasm (guide ATcfc, 2023). This approach is widely employed for the detection of cytokines, enzymes, transcription factors, and signaling pathway components. Of note, the use of viability dyes is critical to monitor live/dead discrimination during staining protocols. Dead cells can interfere with antibody binding and exhibit autofluorescence, ultimately leading to nonspecific labeling and spurious results (Bio Rad Antibodies, n.d.).

Flow cytometry is a technique widely employed in immunology and cell biology studies, enabling a detailed investigation of the immune response and specific cell populations with high accuracy (McKinnon, 2018). In parasitology, flow cytometry has gained increasing relevance both in research and in certain clinical applications. It can be applied to assess parasite load, monitor drug response, and even detect parasite resistance, with potential epidemiological applications in endemic areas (Grimberg, 2011).

Within the context of flow cytometry, it is also possible to implement assays known as Cytometric Bead Array (CBA), which allow the simultaneous quantification of a variety of analytes and, when compared to ELISA, for instance, substantially reduce sample volumes and the time required to obtain results (Medeiros and Gomes, 2019). CBA is based on coupling (binding) a capture molecule (antibody, peptide, protein, among others) to beads (microspheres) internally dyed with different fluorescence intensities, with the purpose of capturing the desired analyte in different samples. Detection and quantification of the analyte(s) are performed through the addition of specific detection antibodies conjugated to a fluorescent molecule for each analyte, which, after acquisition on the flow cytometer, will emit a fluorescent signal corresponding to the concentration of the analyte present in the sample (Morgan et al., 2004). Several studies have already described the use of CBA, such (Ker et al. 2019), which performed a multiplex assay using beads previously coated with two recombinant *Leishmania* antigens: rLci1A and rLci2B. When evaluated against canine serum samples with VL, the assay demonstrated high sensitivity and specificity in distinguishing diseased, vaccinated and healthy dogs.

In cases of cutaneous leishmaniasis, flow cytometry assays have been used in serological studies for diagnosis and treatment monitoring, showing lower specificity than ELISA but higher sensitivity (Pedral-Sampaio et al., 2016). For individuals with malaria, accurate and precise diagnosis is crucial to prevent the progression of the clinical condition of the disease. In this context, flow cytometry has been employed for the analysis of blood infection through the use of antibodies conjugated to fluorochromes specific for nucleic acid staining. Since parasites multiply within erythrocytes, and these cells do not contain DNA, fluorescence is only detected when it binds to the parasite's DNA inside the erythrocyte, thus distinguishing infected cells from healthy ones based on fluorescence intensity. Fluorescence intensity also increases as parasites multiply through mitotic divisions, and this can be analyzed to differentiate the developmental stage of the parasite in blood samples (Janse and Van Vianen, 1994).

In *T. cruzi* infections, flow cytometry has been applied in studies to support the serological diagnosis of Chagas disease, as well as in the follow-up of individuals after treatment with specific drugs, through the use of fixed and labeled *T. cruzi* epimastigote forms for the assessment of IgG reactivity in serum samples, demonstrating high sensitivity and specificity for diagnosis (Matos et al., 2011). Moreover, flow cytometry has also been used as an alternative serological approach for VL identification and as a tool to characterize the humoral response against *Leishmania infantum* in serum samples from VL-HIV coinfected patients (Matos et al., 2011) and presents a higher sensitivity and lower specificity in relation to indirect immunofluorescence in CL infection (Andrade et al., 2010; Rocha et al., 2002).

Among its advantages are high sensitivity and speed. However, it is a more complex and costly technique, requiring specialized equipment and trained personnel. Nonetheless, with the progressive reduction in the cost of cytometers, flow cytometry is expected to play an increasingly important role in the evaluation of parasitic infections, particularly in the development and validation of new drugs and immunotherapies (McKinnon, 2018; Grimberg, 2011). In summary, flow cytometry has increasingly emerged as a versatile tool with broad applicability in the diagnosis of parasitic diseases, enabling the detection and characterization of infections and contributing to significant advances in both research and clinical practice.

## 6 Molecular diagnosis

Molecular testing refers to the analysis of biological molecules such as DNA, RNA, and proteins from clinical samples, including blood or tissue, to aid in disease detection and prediction, for example the Western Blotting and PCR tests. In this context, recent advances in molecular testing methods are reshaping the field of infectious disease diagnostics. Over the past decade, nucleic acid–based technologies have increasingly complemented conventional approaches such as culture, antigen detection, and serology for the identification and epidemiological characterization of infectious microorganisms. Until recently, molecular diagnostics in infectious diseases were largely restricted to the detection of slow-growing, fastidious, or non-cultivable pathogens. However, innovations in testing platforms—particularly automated nucleic acid extraction systems and rapid polymerase chain reaction (PCR)–based target detection formats—have expanded their applicability, making molecular diagnostics feasible in a broad range of clinical laboratories and for a wide spectrum of common pathogens (Wolk et al., 2001).

### 6.1 Polymerase chain reaction (PCR)

PCR is one of the most widely employed methodologies in diagnostic laboratories, as it enables the rapid generation of results that guide appropriate therapeutic decisions in response to detected infections. The process mimics the natural duplication of DNA *in vivo*, albeit with key differences, including the exclusive use of DNA polymerase (Khehra et al., 2025).

The reaction requires essential components: primers, DNA polymerase, PCR buffer, cofactors, deoxynucleotide triphosphates (dNTPs), and water. The thermostable enzyme responsible for synthesizing the DNA strand is Taq polymerase, originally isolated from the thermophilic bacterium *Thermus aquaticus*. Primers act as specific initiators for the selected target sequence. The PCR buffer provides an optimized environment, containing cofactors such as MgCl_2_, while the dNTPs—thymine (T), adenine (A), cytosine (C), and guanine (G)—serve as the building blocks for DNA synthesis. When present in the correct proportions, these components ensure efficient enzyme activity and overall reaction performance (Garibyan and Avashia, 2013).

**Figure 7:**
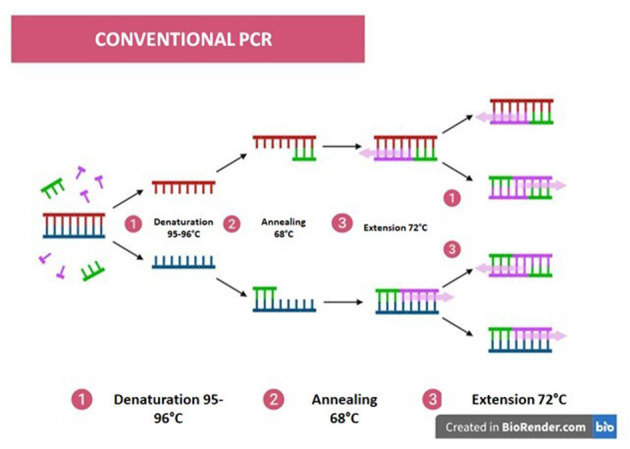
Schematic of the steps of Conventional PCR: (1) Denaturation (95–96 °C), separation of DNA strands; (2) Annealing (≈68 °C), ligation of primers to template strands; (3) Extension (72 °C), synthesis of the new DNA strand by DNA polymerase. Image: Produced using BioRender.

The PCR cycle consists of three major steps: denaturation at 94 °C −95 °C to separate the DNA strands; annealing at a reduced temperature to allow proper primer binding; and extension at 68 °C −72 °C, depending on the kit used, to synthesize the new DNA strand. An additional 5-min extension step at the end of the cycles is commonly included to ensure completion of amplification (Figure 7) (Khehra et al., 2025).

Conventional PCR requires that the patient's sample DNA be extracted using column-based methods, phenol/chloroform, or magnetic beads. It is worth noting that at low concentrations, there is an increased likelihood of false negatives and/or nonspecific results, in addition to the potential contribution of inhibitors to reaction failure, as well as interference from proteins and salts (Khehra et al., 2025). Therefore, this reaction relies on several factors to be properly executed. Its application in diagnostics is still employed, particularly in regions lacking the infrastructure to routinely perform different PCR modalities, since the resource requirements and higher costs of reagents for other techniques often make the implementation of newer methods unfeasible in more remote settings.

New PCR modalities have been developed, including conventional PCR, qPCR (Quantitative), Nested PCR, among others. Conventional PCR remains the most widely used in many laboratories due to its low cost and reliable amplification at a relatively affordable level, in addition to its ability to amplify larger targets. The method is followed by a qualitative analysis, as it indicates the presence or absence of a specific pathogen through the appearance of bands in electrophoresis corresponding to the gene within the target DNA (Garibyan and Avashia, 2013). Comparing to this, quantitative PCR, or qPCR, enables not only detection but also the estimation of absolute or relative quantification of the number of amplified DNA copies of the target gene (Arya et al., 2005).

#### 6.1.1 New PCR modalities

Nested PCR consists of one of the variations of Conventional PCR that allows for increased sensitivity and specificity of the methodology. This is achieved by the use of internal primers to enhance accuracy, especially when dealing with samples presenting low DNA concentrations, such as those derived from fixed tissues or blood (Green and Sambrook, 2019; Uppal et al., 2014).

In addition to the initial primers that target the gene of interest, it is necessary to design internal primers so that, during the second reaction, the “nested” step occurs, where the initially amplified DNA is itself used as the template DNA in the subsequent reaction. The use of these internal primers is essential to obtain amplicons (fragments of DNA amplified with higher specificity), avoiding the appearance of nonspecific bands (Viana et al., 2016).

It is considered a valid diagnostic tool for detecting submicroscopic infections, since patients may be asymptomatic yet infected, with parasite concentrations below the detection threshold. A relevant example is infection by *Plasmodium* spp., in which low DNA concentrations are often observed in submicroscopic cases, as well as in infections caused by the parasite itself. Furthermore, Nested PCR is valuable for accurate species-level diagnosis, since therapeutic regimens vary depending on the infecting species. It is crucial to highlight that in infections caused by *P. vivax*, dormant hypnozoite forms may develop, which must be treated with tafenoquine or primaquine. This aspect makes accurate diagnosis and therapeutic approach relevant (Fitri et al., 2022; Snounou and Beck, 1998; Snounou et al., 1993).

The qPCR, or real-time PCR, represents a more advanced and faster methodological approach compared to Conventional PCR, as this variation of the technique enables the analysis of results to be performed in a single step. This allows for both the detection and quantification of the same amplified target gene corresponding to a given pathogen within a sample (Arya et al., 2005), while simultaneously permitting the monitoring of the PCR process throughout the reaction (Artika et al., 2022). The ability to track amplification in real time and to quantify parasite load ensures that diagnosis can be carried out more rapidly while maintaining high accuracy.

In this modality, fluorescent probes and intercalating agents are employed to detect the target gene, enabling quantification as well as the analysis of genetic variations after sequencing of the sample. Both probe-based approaches, such as TaqMan, and DNA-intercalating dyes, such as SYBR Green, can be used (Arya et al., 2005; Liu et al., 2006; Ma et al., 2021). It is important to emphasize that the distinction between probes and intercalating agents lies in their specificity, which is particularly relevant for diagnostic purposes. Unlike probes, intercalating dyes detect both specific and nonspecific amplified products (Liu et al., 2006). In summary, at the level of data analysis and curve interpretation, it must be highlighted that this assay does not quantify the total DNA concentration, but rather the number of copies of the amplified sequence. Therefore, defining the cycle threshold (Ct)—the number of amplification cycles required for fluorescence to surpass the threshold and rise above the baseline—is critical (Artika et al., 2022; Ma et al., 2021).

The distinction between absolute and relative quantification lies in the generation of a standard curve. Absolute quantification requires a calibration curve, which can be constructed using serial dilutions of samples such as plasmids or synthetic oligonucleotides, thereby enabling the estimation of copy numbers and quantification of the target gene. This process is especially relevant for assessing parasite load in infectious and parasitic diseases. In contrast, relative quantification does not necessarily require a standard curve but relies instead on comparative analysis between the expression of the target gene and an endogenous reference gene (Dhanasekaran et al., 2010; Harshitha and Arunraj, 2021).

The LAMP PCR consists of a variation of Conventional PCR that allows its use mainly in field situations and in remote areas, since its cycling can be carried out at a constant temperature, ranging between 60 °C and 65 °C. In this reaction, a water bath is required, without the need for an expensive and difficult-to-transport thermocycler, making this technique a promising molecular diagnostic approach for regions with endemic diseases, especially those with little or no infrastructure for performing molecular biology assays (Srivastava and Prasad, 2023; Yang et al., 2024).

It is also a promising diagnostic methodology regarding the constant monitoring of cases, including those considered asymptomatic and/or submicroscopic, which fosters the implementation of active genomic surveillance. This is a strictly necessary demand both for the recognition of infectoparasitic cases and for the analysis of genetic diversity and how these alterations contribute to immune escape (García-Bernalt Diego et al., 2021).

Multiplex PCR consists of a molecular biology methodology that allows the amplification of different genes from distinct pathogens, meaning that within a single reaction it is possible to identify the origin of the infection and determine the etiological agent. This technique is particularly relevant in endemic areas of parasitic infections and other diseases, mainly because it enables the identification of the etiological agent in a short period of time, thereby allowing the timely initiation of treatment (Llewellyn et al., 2016). It is possible to employ additional pairs of primers to perform the reaction; however, it is strictly relevant that they are well designed and possess annealing temperatures that can be standardized and adapted to the different primer pairs. The main limitation of the technique lies in obtaining nonspecific results due to inadequate interaction between the primers. This occurs because primers may form dimers with each other or even self-dimers, which can inhibit the amplification of the target sequences or generate nonspecific products (de Korne-Elenbaas et al., 2025).

Multiplex PCR plays a relevant role in clinical diagnosis because it enables the accurate identification of the infectious agent, as well as guiding the therapeutic regimen in a single reaction. In this approach, a sample can be tested for different diseases, including pathogens hypothesized during anamnesis and clinical suspicion, even if they are not of infectoparasitic origin. However, this technique presents greater difficulty with standardization due to differences in the characteristics of the primers used for the distinct molecular targets (McAuliffe et al., 2013). Due to possible erroneous interactions among the designed primers, assertive experimental planning is required, since the targets may be quite distinct and must be carefully analyzed for the reaction to work properly, that is, to achieve the development of a functional and complete panel.

#### 6.1.2 Applications of molecular diagnostic tools in malaria, Chagas disease, and leishmaniasis

In malaria diagnosis, molecular diagnosis is essential for accurately identifying the infecting species and guiding appropriate treatment. The Nested PCR technique, described by Snounou and colleagues, is a variation of conventional PCR with enhanced specificity, thereby improving diagnostic accuracy (Snounou and Beck, 1998; Snounou et al., 1993). In endemic regions with limited infrastructure, LAMP-PCR emerges as a viable alternative. This technique operates at a constant temperature (~60 °C) and enables both qualitative and quantitative analyses (Fitri et al., 2022; Feleke et al., 2021). Although sensitivity and specificity may vary, its application is particularly relevant since malaria rapid diagnostic tests (MRDTs) have reduced sensitivity in cases of low parasitemia or subclinical infections. Therefore, molecular biology tools represent an effective strategy to complement clinical diagnosis, and can be performed using samples of whole blood, urine, serum, plasma, and saliva, but DNA extraction from whole blood is commonly performed due to the development of the infection and its stages, especially the blood stage (Feleke et al., 2021; Danwang et al., 2021; Roth et al., 2016).

For leishmaniasis infections, accurate diagnosis is a crucial step in managing the disease, which can present in cutaneous or visceral forms. For the tegumentary form of leishmaniasis, molecular diagnosis can be performed using samples derived from biopsies, scraping of the lesion, aspiration puncture of the lesion, and swabs. In contrast, visceral leishmaniasis requires splenic aspiration, bone marrow, lymphatic, peripheral blood, serum, skin, urine and buffy coat (de Ruiter et al., 2014; de Vries and Schallig, 2022; Saab et al., 2015; Srividya et al., 2012). As with other parasitic infections, conventional PCR is a highly relevant methodological approach for performing sensitive and specific diagnostic tests. Quantitative PCR (qPCR), in particular, allows for the quantification and monitoring of parasite load, which is critical for case reporting, epidemiological surveillance, and evaluating patient response to therapy. In this infection, Nested PCR, conventional PCR, PCR-RFLP, and qPCR are all useful for target amplification and for discriminating the etiological agent, either through polymorphism analysis or simple detection of kDNA (Gow et al., 2022). For amplification and differentiation of circulating species that may cause infection, the 18S rRNA gene of *Leishmania* has proven to be the most sensitive and specific target, allowing precise species discrimination and enabling a more targeted therapeutic approach (León et al., 2017). Moreover, other targets has also been explored, for an example, hsp70 and ITS1 (Gritti et al., 2023; Mohammadi Manesh et al., 2025).

For Chagas disease, molecular diagnosis is primarily through PCR (or its quantitative variants qPCR and LAMP) and it's a highly sensitive method that detects *T. cruzi* DNA, especially in the chronic phase when parasite levels are low. PCR offers greater sensitivity than microscopy for detecting the parasite during the acute phase and is also used for post-treatment monitoring to assess cure. Therefore, for molecular diagnosis of Chagas disease, clinical samples of peripheral blood, líquor, biopsy and tissue samples can be used (Bautista-Lopez and Ndao, 2024; Lopez-Albizu et al., 2020; Moreira et al., 2023; Schijman, 2023) However, limitations in standardization and availability exist, and it is often performed in specialized laboratories, particularly when serological tests yield inconclusive results (Pascual-Vázquez et al., 2023).

### 6.2 Western blotting

Western blotting (WB), also known as immunoblotting, is a well-established and widely used technique in molecular biology and proteomics (Mahmood and Yang, 2012). This method allows the detection, identification, and quantification of specific proteins within a biological sample. The term “blotting” refers to the transfer of proteins, previously separated by gel electrophoresis, onto a membrane (typically nitrocellulose), where they are subsequently recognized by specific antibodies (Towbin et al., 1979; Burnette, 1981). WB is notable for its high sensitivity and specificity, enabling the detection of proteins even at very low concentrations (Kurien and Scofield, 2006). Beyond its widespread use in scientific research, Western blotting is also applied in the diagnosis of various diseases, including infections and genetic disorders (Mahmood and Yang, 2012).

According to (Heidari et al. 2019), Western blotting was evaluated for its diagnostic performance by detecting a specific pattern of immunodominant *Leishmania infantum* proteins using sera from patients with visceral leishmaniasis. The results showed that the technique could identify multiple proteins specifically recognized by patient antibodies, confirming its high sensitivity and specificity. Thus, Western blotting proved extremely useful for confirming suspected cases or those with inconclusive serological results, making it a valuable complementary diagnostic tool in laboratories with adequate infrastructure. As reported by (Ascanio et al. 2024), although Western blotting demonstrates high accuracy in confirming *Trypanosoma cruzi* infection, operational limitations—such as high cost, technical complexity, and limited availability—restrict its use to reference laboratories and research centers. In this context, the method is essential for resolving cases with discordant serological results but is not routinely implemented in conventional clinical laboratories.

For malaria diagnosis, this technique proved effective in detecting different *Plasmodium* species and has been considered a useful tool for serological diagnosis, mass screening in endemic regions, and safety testing in blood transfusions in areas with prevalent *P. vivax* malaria (Son et al., 2001). In the context of Chagas disease diagnosis, Western blotting has been described as a supplemental method, particularly useful when analyzing samples from children under 5 years old from various regions of Brazil (Frade et al., 2011).

In patients co-infected with other pathogens, especially *Leishmania* species, cross-reactivity often limits the specificity of serological tests, representing a major challenge in areas endemic for both *T. cruzi* and *Leishmania* spp. Although the use of recombinant antigens can partially reduce cross-reactions, it does not fully resolve the issue (Riera et al., 2012). Western blotting offers an advantage over conventional serological techniques because it allows the identification of antibodies targeting distinct polypeptide fractions within complex parasite antigen mixtures. Consequently, this approach can provide higher specificity and improved performance compared to other immunological methods (Riera et al., 2012).

## 7 Conclusion

It can be concluded that infectious diseases represent a serious public health problem, regardless of whether they are classified as neglected or not. In this context, diagnostic tests play a crucial role in the identification, control, and management of parasitic diseases. In addition to enabling clinical confirmation of infections, they provide essential epidemiological data to guide public health policies, prevention strategies, and surveillance programs. The use of laboratory methods, including serological and molecular approaches, should be guided not only by the patient's clinical manifestations and medical history but also by epidemiological factors such as geographic location and travel history, allowing for effective screening of prevalent diseases in a given region. Thus, the integration of clinical and epidemiological information with sensitive and specific diagnostic techniques forms the foundation for early detection, monitoring, and control of infections in endemic areas, reinforcing the importance of diagnosis as a strategic tool in public health. Additionally, factors such as resource availability in endemic areas and the combined or individual use of serological and molecular tests should be considered when selecting the appropriate diagnostic method.
